# TRUST: A toolkit for TEE-assisted secure outsourced computation over integers

**DOI:** 10.1016/j.fmre.2025.08.017

**Published:** 2025-11-01

**Authors:** Bowen Zhao, Jiuhui Li, Cheng Qiao, Jia-Nan Liu, Qingqi Pei, Yulong Shen

**Affiliations:** a Guangzhou Institute of Technology, Xidian University, Guangzhou 510555, China; b State Key Laboratory of Public Big Data, Guizhou University, Guiyang 550025, China; c Cyberspace Institute of Advanced Technology, Guangzhou University, Guangzhou 510555, China; d School of Computer Science and Technology, Dongguang University of Technology, Dongguan 523808, China; e State Key Laboratory of Integrated Service Networks, Xidian University, Xi’an 710126, China

**Keywords:** Secure computing, Privacy protection, TEE, Homomorphic encryption, Data trading

## Abstract

Secure outsourced computation (SOC) provides secure computing services by taking advantage of the computation power of cloud computing and the technology of privacy computing (e.g., homomorphic encryption). Expanding computational operations on encrypted data (e.g., enabling complex calculations directly over ciphertexts) and broadening the applicability of SOC across diverse use cases remain critical yet challenging research topics in the field. Nevertheless, previous SOC solutions frequently lack the computational efficiency and adaptability required to fully meet evolving demands. To this end, in this paper, we propose a toolkit for TEE-assisted (Trusted Execution Environment) SOC over integers, named TRUST. In terms of system architecture, TRUST falls in a single TEE-equipped cloud server only through seamlessly integrating the computation of REE (Rich Execution Environment) and TEE. In consideration of TEE being difficult to permanently store data and being vulnerable to attacks, we introduce a (2, 2)-threshold homomorphic cryptosystem to fit the hybrid computation between REE and TEE. Additionally, we carefully design a suite of SOC protocols supporting unary, binary and ternary operations. To achieve applications, we present SEAT, secure data trading based on TRUST. Security analysis demonstrates that TRUST enables SOC, avoids collusion attacks among multiple cloud servers, and mitigates potential secret leakage risks within TEE (e.g., from side-channel attacks). Experimental evaluations indicate that TRUST outperforms the state-of-the-art and requires no alignment of data as well as any network communications. Furthermore, SEAT is as effective as the Baseline without any data protection.

## Graphical abstract

**Figure:**
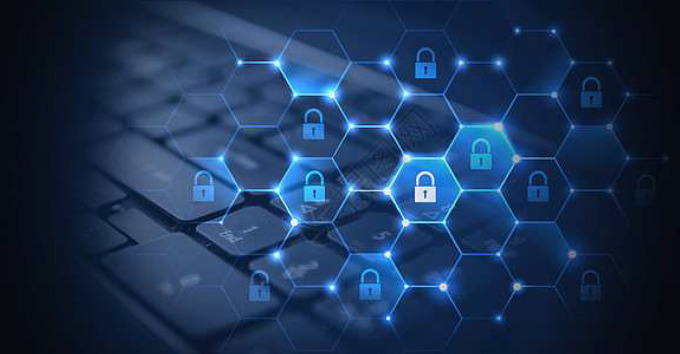

## 1 Introduction

Outsourcing computation allows a user with limited resources to outsource operations (or rather, computations) over data to a cloud server. Secure outsourced computation (SOC) ensures computations over data without disclosing the data itself [1]. In other words, SOC provides not only convenient computing services for the user with limited resources, but also privacy protection for the outsourced data. Broadly speaking, many types of secure operations can be considered as SOC, for instance, searchable encryption [2], private set intersection [3], secure training or inference for machine learning [4], and secure computation as a service [5]. Thus, SOC can be applied to various fields including search, product recommendation, artificial intelligence, production optimization, etc.

Technically, a cloud server providing SOC services usually adopts privacy computing technologies to safeguard the privacy of outsourced data. Homomorphic encryption (HE), secure multi-party computation (MPC), and trusted execution environment (TEE) [6] are common privacy computing technologies. HE allows operations over encrypted data (also known as ciphertexts) directly and accesses no decryption key [7]. MPC enables multiple parties to jointly perform an operation and safeguards each party’s inputs [8]. SOC solutions based on multiple servers usually rely on MPC. Both HE and MPC achieves SOC through software method, while TEE is through a hardware method. TEE creates an enclave offering hardware-based memory encryption for code and data, in which no unauthorized entities from outside the enclave can access inside code or data [9]. Such capabilities position TEE as a promising hardware-based approach to secure computation, achieving CPU-level performance and effectively bridging the gap between academic research and industrial adoption in the field.

Existing SOC solutions remain constrained by limited computational operations and often assume no collusion among cloud servers. On the one hand, SOC based on HE usually supports secure addition, secure multiplication, or both. However, in practice, a user requires more operations (e.g., secure comparison) offered by SOC to handle outsourced data. On the other hand, although SOC based on MPC offers more secure operations, it requires at least two cloud servers and assumes that there is no collusion between cloud servers. In this case, more cloud servers mean more deployment costs. Additionally, more network communications and extra data alignment operations—ensuring the synchronization of necessary computation data across multiple servers are required to enable secure computations. Arguably, the assumption of no collusion may be too strong, as it is difficult to determine whether cloud servers launch the collusion attack or not. SOC based on TEE enriches computational operations and avoids the collusion attack launched by multiple cloud servers [10]. Unfortunately, any enclave holds limited storage space, and its storage is Random Access Memory (RAM) [11]. Thus, it is not trivial to permanently store and process outsourced data in an enclave. Furthermore, TEE is not always trusted because side-channel attacks are high likely to expose inside code and data in the enclave [12].

In order to mitigate the above limitations faced by SOC, in this work, we concentrate on devising a hybrid SOC framework by integrating advantages from the software-based method and the hardware-based method. Intuitively, this framework is confronted with several challenges. The **first** one is to balance the software method and the hardware method, and then eliminate collusion attacks. The software-based method and the hardware-based method follow entirely different designs to achieve SOC. A basic operation is supported by the former but not by the latter, and vice versa. The **second** one is to perform secure computations in the absence of a fully trusted enclave—one that honestly executes all required operations and never attempts to access unauthorized data. Due to the degradation of trust in enclave, some excellent features provided by the fully trusted enclave cannot be adopted. Thus, this significantly limits the functionality of the enclave. The **last** one is to enrich secure operations falling within this framework. To eliminate the collusion attack, this framework should not utilize MPC to enable SOC, which restricts secure operations. Moreover, the enclave in this framework fails to enrich secure operations by reason of trust concerns. Worse, HE-based SOC only achieves simple secure operations.

In response to the aforementioned challenges, in this work, we propose TRUST,^1^ a toolkit for TEE-assisted SOC over integers. In brief, the proposed TRUST employs a single TEE-equipped cloud server, in which a Rich Execution Environment (REE) of the cloud server cooperates with TEE to provide SOC services. A (2, 2)-threshold Paillier cryptosystem [13] is introduced in TRUST to construct SOC protocols. After that, we come up with secure data trading based on TRUST, called SEAT^2^ to explore the application of SOC. Our innovations in this work involve three fronts.

• **Practical SOC framework**. We propose a practical SOC framework that relies on a single TEE host, where the TEE host calls a software-based method (i.e., the threshold Paillier cryptosystem [13]), and the enclave of TEE offers hardware-based capabilities. The TEE host and the enclave serve as the two parties of SOC and jointly perform SOC protocols. Particularly, the proposed SOC framework does not require a fully trusted enclave.
• **Rich SOC operations**. Following the proposed framework and assumption, we carefully design a suite of underlying SOC protocols encompassing unary, binary and ternary operations.
• **Feasible SOC application**. We apply the proposed TRUST to data trading and present a solution SEAT, which not only fulfills the outsourced task requirements but also effectively avoids data resale and privacy leakage.

The rest of this work is organized as follows. We briefly review related work in Section 2. Subsequently, Section 3 presents the preliminaries essential for constructing TRUST. After that, we present the system model and the threat model of TRUST and elaborate on its design in Sections 4 and 5, respectively. In Sections 6 and 7, the application and the security of TRUST are given, respectively. We extensively evaluate the efficiency and effectiveness of both TRUST and SEAT in Section 8. Finally, we conclude this work in Section 9.

## 2 Related work

In this section, we briefly review software-based and hardware-based approaches to SOC.

### 2.1 Software-based SOC

HE-based solutions (e.g., [14], [15], [16]) mainly focus on enabling a wide range of operations over ciphertexts (e.g., arithmetic, logical) and supporting diverse numerical representations (e.g., integers, floating-point numbers) [1], [17]. To support the generation of private recommendations, Erkin *et al*[14]. proposed a secure comparison protocol for multiple packed values. This protocol relies on a semi-trusted server with decryption capabilities that jointly employs two additively homomorphic cryptosystems, including Paillier [18] and Damgrd-Geisler-Krȹigaard [19]. The work [15] designed a novel secure k-nearest neighbor (SkNN) protocol using based on the Paillier cryptosystem that enables privacy-preserving searches on encrypted cloud data. Although HE supports computations over ciphertexts, solutions such as [15], [16] suffer from security issues, as they assign the entire decryption key to a server, which creates a single point of failure. Moreover, other solutions (e.g., somewhat HE [20] and fully HE [21]) either lack support for arbitrary computations or remain impractical due to substantial computational overhead.

The integration of a twin-server architecture with HE makes arbitrary operations over ciphertexts possible, while simultaneously eliminating the single point of security failure. Following this paradigm, several schemes [7], [13], [22], [23] employed a (2,2)-threshold Paillier cryptosystem to expand the set of operations available over ciphertexts. In more detail, Liu et al. [23] proposed POCF, a solution that enriches ciphertext operations by distributing Paillier private keys between two servers. This design ensures neither server can independently access raw data, enabling the clouds to complete SOC tasks through collaborative decryption. Likewise, the work [7] implemented SOCI, a toolkit for SOC on integers based on a twin-server architecture with threshold Paillier cryptosystem, providing more efficient secure multiplication and secure comparison protocols than prior work [23]. Furthermore, the recently proposed SOCI^+^ [13] demonstrates a substantial performance improvement over its predecessor, SOCI [7]. This advancement is primarily driven by a more efficient threshold Paillier variant, namely FastPaiTD, and an offline-online mechanism that supports pre-computation. However, all the aforementioned work fundamentally relies on the non-collusion assumption between the twin-server, which falls short of real-world requirements.

MPC-based solutions, which are more inclined to extend SOC for specific applications, are represented by Yao’s garbled circuits (GC) [24] and secret sharing (SS) [25]. Blanton et al. [26] presented a secure sequence comparison scheme (i.e., edit distance) that employs GC in a novel non-black-box manner with two servers, making it suitable for privacy-preserving DNA alignment and other string matching tasks. An iris identification scheme is proposed in the work [27], enabling secure iris matching on untrusted servers via predicate encryption in a single-server model or SS in a multi-server model. Additionally, ABY [28] and ABY 2.0 [29] frameworks employed SS to implement mixed protocols for secure two-party computation, integrating arithmetic and boolean operations. Although these schemes facilitate distributed privacy-preserving computation and can support arbitrarily complex functions, they demand exorbitant storage and communication resources and always rely on the non-collusion assumption among parties.

### 2.2 Hardware-based SOC

TEE-based solutions utilizing commodity hardware (e.g., Intel® SGX, ARM TrustZone) drive advances in secure computing research and trusted computing applications. Representative examples include outsourced computation [10], [30], credential migration [31], root-of-trust design [32], formal verification framework [33], electronic healthcare data sharing [34], robust federated learning [35], access control [36], data trading [37], among others.

Liu et al. [30] presented a SOC scheme, integrating blockchain, Intel® SGX, and smart contracts under the assumption of a semi-honest server. This scheme treats the enclave as a black box, i.e., a fully trusted enclave. Specifically, encrypted data is transmitted exclusively to the enclave, where tasks are executed over plaintext, and the results are signed with the enclave’s private key before being output. While its core advantage lies in ensuring data security and result credibility, it overlooks potential security vulnerabilities in TEE. Moreover, the deployment of extensive processing tasks on-chain introduces notable computational costs. The work [10] designed a fast processing toolkit on a single cloud server supporting secure integer and floating-point computations. An interesting feature of this work is that key and data shares are distributed across multiple trusted processing units (TPUs) to enable distributed decryption and collaborative computation, while effectively resisting side-channel attacks against TEEs. However, the use of multiple TPUs introduces higher deployment and computation costs, as well as more frequent inter-server communication rounds. Dai et al. [37] put forward SDTE, a blockchain-based secure data trading system utilizing TEE and AES-256 symmetric encryption. Within this system, TEE establishes secure channels with buyers and sellers via authentication technologies and acts as a secure intermediary, where all data decryption and processing are performed within the enclave. Obviously, this system model is also inadequately considered, neglecting the vulnerability of TEE to side-channel attacks.

More than the aforementioned work, many existing TEE-based solutions also face practical limitations, including restricted computational resources and poor scalability, which hinder their applicability in complex, resource-intensive scenarios. TEE is not bullet-proof and has been successfully attacked numerous times in various ways, as shown in [38], [39], [40]. Undoubtedly, treating TEE as an unconditionally trusted black box for direct plaintext computation or master key management represents a flawed security model, which can lead to catastrophic privacy leakage.

## 3 Preliminaries

In this section, we introduce the threshold Paillier cryptosystem and TEE, which serve as the foundation for the design of TRUST.

### 3.1 FastPaiTD

FastPaiTD proposed by the work [13], a (2,2)-threshold Paillier cryptosystem, serves as the preliminaries of our proposed TRUST. FastPaiTD involves 5 probabilistic polynomial-time algorithms listed as follows.

1) KGen (key generation): KGen takes a secure parameter κ (e.g., κ=112) as input and outputs a public/private pair (pk,sk) and two partially private keys $\left(sk_{1},sk_{2}\right)$. Formally, $\mathrm{KGen}\left(1^{\kappa }\right)\to \left(pk,sk,sk_{1},sk_{2}\right)$. Specifically, pk=(N,h), sk=α, and $\left(sk_{1},sk_{2}\right)$ satisfy $sk_{1}+sk_{2}=0\text{mod}2\alpha$ and $sk_{1}+sk_{2}=1\text{mod}N$. More details about the above parameters can be found in the work [13]. Note that we set $sk_{1}$ as a σ-bit (e.g., σ=128) random number and compute $sk_{2}=2\alpha \cdot {\left(2\alpha \right)}^{-1}\text{mod}N-sk_{1}$.

2) Enc (encryption): Enc takes as input a message $m\in Z_{N}$ and the public key pk, and outputs a ciphertext ⟦m⟧ as follows:

(1) $$⟦m⟧\leftarrow {\left(1+N\right)}^{m}\cdot {\left(h^{r}\;\text{mod}\;N\right)}^{N}\text{mod}\;N^{2},$$

where r is a 4κ-bit random number. Formally, Enc(pk,m)→⟦m⟧.

3) Dec (decryption): Dec takes as input ⟦m⟧ and the private key sk and outputs a message m as follows:

(2) $$m\leftarrow \left(\frac{⟦m{⟧}^{2\alpha }\;\text{mod}\;N^{2}-1}{N}\text{mod}\;N\right)\cdot {\left(2\alpha \right)}^{-1}\text{mod}\;N.$$

Formally, Dec(sk,⟦m⟧)→m.

4) PDec (partial decryption): PDec takes as input ⟦m⟧ and a partially private key $sk_{i}$ (i∈{1,2}) and outputs a ciphertext as follows:

(3) $${\left[m\right]}_{i}\leftarrow ⟦m{⟧}^{sk_{i}}\;\text{mod}\;N^{2}.$$

Formally, $\mathrm{PDec}\left(sk_{i},⟦m⟧\right)\to {\left[m\right]}_{i}$ for i∈{1,2}.

5) TDec (threshold decryption): TDec takes as input ${\left[m\right]}_{1}$ and ${\left[m\right]}_{2}$ and outputs a message m as follows:

(4) $$m\leftarrow \frac{({\left[m\right]}_{1}\cdot {\left[m\right]}_{2}\;\text{mod}N^{2})-1}{N}\;\text{mod}\;N.$$

Formally, $\mathrm{TDec}({\left[m\right]}_{1},{\left[m\right]}_{2})\to m$.

Note that FastPaiTD supports additive homomorphism and scalar-multiplication homomorphism. Specifically,

• Additive homomorphism: Given two ciphertexts $⟦m_{1}⟧$ and $⟦m_{2}⟧$, $\mathrm{Dec}(sk,⟦m_{1}+m_{2}\text{mod}N⟧=\mathrm{Dec}\left(sk,⟦m_{1}⟧\cdot ⟦m_{2}⟧\text{mod}N^{2}\right)$ holds.
• Scalar-multiplication homomorphism: Given a ciphertext ⟦m⟧ and a constant $c\in Z_{N}$, $\mathrm{Dec}\left(sk,⟦c\cdot m\text{mod}N⟧\right)=\mathrm{Dec}\left(sk,⟦m{⟧}^{c}\text{mod}N^{2}\right)$ holds. Particularly, when c=−1, $\mathrm{Dec}(sk,⟦m{⟧}^{c}\text{mod}N^{2})=-m$ holds.

### 3.2 Trusted execution environment (TEE)

TEE performs confidential computing by providing a secure enclave within the processor, specifically designed to protect sensitive code and data from any potentially malicious entities residing in the system (including the kernel, hypervisor, etc.) [9]. Notably, TEE-based secure computation has attracted significant interest from researchers in both academia and industry over the past two decades (e.g., [41], [42], [43], [44]), thanks to its three essential features.

• Attestation [45]. Attestation is a method of establishing trust that employs cryptographic proofs to verify both the identities of enclaves and the integrity of the software running within them. There are two primary forms of attestation. Local attestation enables mutual verification between enclaves residing on the same physical platform. Remote attestation allows a client to verify the correct and unmodified code is running inside a specific enclave on a remote server.
• Isolated execution [46]. TEE enforces robust isolation by partitioning the host memory into a secure, trusted region and a non-secure, untrusted region. The trusted region, known as an enclave, offers both confidentiality and integrity guarantees of its internal code and data. In contrast, the untrusted region, called REE, remains entirely accessible to the operating system. Specifically, sensitive data within an enclave is stored in the EPC (Enclave Page Cache), a hardware-protected memory region allocated in system DRAM (Dynamic Random Access Memory) during the bootstrapping phase. The code loaded inside an enclave can access both EPC and non-EPC memory, while all external programs are restricted from accessing the EPC. All interactions between REE and TEE are strictly controlled through programmer-defined entry points.
• Sealing and unsealing [45]. Sealing and unsealing is a mechanism that allows a TEE to securely store and retrieve a secret in non-volatile external storage. On the one hand, TEE can encrypt and store secrets using a CPU-derived key bound to its identity (e.g., Intel® SGX’s MRENCLAVE), referred to as sealing. On the other hand, it can retrieve and securely decrypt these secrets from storage, referred to as unsealing.

Although TEE safeguards processed data by encrypting both incoming and outgoing data flows, research has shown that it remains vulnerable to various side-channel attacks, including those targeting page tables [38], [47], DRAM [39], [48], [49], and cache [40], [50], [51].

## 4 System model and threat model

**Fig. 1:**
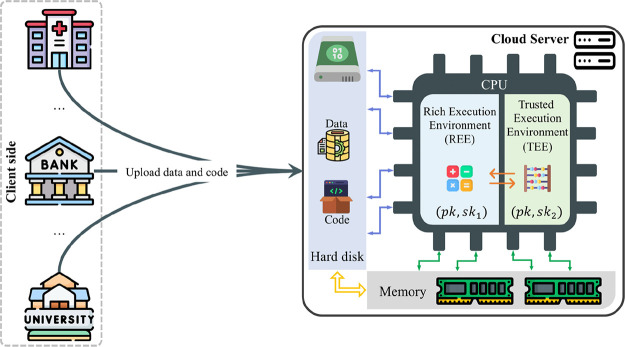
**System model of TRUST**.

The system model formulates the entities within a system and their abilities. As depicted in Fig. 1, our system model contains two entities, that is, a client and a cloud server, where the cloud server is equipped with a TEE chip and segregates code execution into two regions, i.e., the REE and the TEE.

• **Client.** The client is a user who requires SOC over ciphertexts. The user may be, for example, a hospital, bank, university, etc. To this end, the user initializes the (2,2)-threshold Paillier cryptosystem and generates the public key pk along with two partial private keys, $sk_{1}$ and $sk_{2}$. Furthermore, the user assigns $\left(pk,sk_{1}\right)$ to the REE and transmits $\left(pk,sk_{2}\right)$ to the TEE via a secure channel. The user stores ciphertexts and code on the hard disk of the cloud server.
• **Cloud server.** The cloud server must be equipped with a TEE on its CPU and provide SOC services to the user via its REE and TEE. Specifically, the REE initiates a computation and collaborates with the TEE to obtain the corresponding result.

The threat model assumes the attack capabilities of all entities in the system. In TRUST, we suppose the client is fully trusted, whereas the TEE is only partially trusted because its internal state (including code and data) may not be robust enough to prevent all side-channel attacks. Consequently, the core requirement for the TEE in our design is to ensure the integrity of the enclave’s internal state, while focusing on a software-hardware hybrid approach (i.e., applying HE within TEE) to strengthen overall security. The REE is semi-honest, meaning it follows the prescribed protocols but attempts to obtain the client’s data and calculation results. Notably, any adversary is allowed to corrupt REE or TEE, but never both at the same time.

## 5 TRUST design

In this section, we first formalize the SOC of TRUST. After that, a suite of SOC protocols integrated into TRUST is introduced.

### 5.1 Problem formulation

Suppose encrypted outsourced data $⟦m_{1}⟧,⟦m_{2}⟧,\ldots ,⟦m_{n}⟧$ require performing SOC, denoted by F, where n is the number of operands and n≥1. Our proposed TRUST assumes $f_{r}$ and $f_{t}$ denote the computation over REE and TEE, respectively. Particularly, TRUST formalizes the SOC $F(⟦m_{1}⟧,\ldots ,⟦m_{n}⟧)$ as follows.

Definition 1 Suppose a SOC be denoted by $F(⟦m_{1}⟧,\ldots ,⟦m_{n}⟧)$, where n is the size of outsourced data, TRUST formulates it as a two-party computation between REE and TEE denoted by

(5) $$F\left(⟦m_{1}⟧,\ldots ,⟦m_{n}⟧\right)\;\;\;\Leftrightarrow \;\;\;f_{t}\left(key_{t},f_{r}\left(key_{r},⟦m_{1}⟧,\ldots ,⟦m_{n}⟧\right)\right).$$

$key_{r}$ and $key_{t}$ indicate the key of REE and TEE, respectively. Note that $f_{t}$ can be omitted when $f_{r}$ enables computations required by F.

From Eq. (5), we see that only REE accesses outsourced data $⟦m_{1}⟧,\ldots ,⟦m_{n}⟧$ directly, while TEE takes the output of REE as input. According to the system model of TRUST, $key_{r}$ and $key_{t}$ denote $\left(pk,sk_{1}\right)$ and $\left(pk,sk_{2}\right)$, respectively, thus, the output of $f_{r}$ is encrypted data. Note that when n=1, $F(⟦m_{1}⟧)$ means a unary operation (e.g., absolute value, factorial). $F(⟦m_{1}⟧,⟦m_{2}⟧)$ (n=2) indicates a binary operation (e.g., addition, multiplication, comparison), while $F(⟦m_{1}⟧,⟦m_{2}⟧,⟦m_{3}⟧)$ (n=3) denotes a ternary operation (e.g., ternary conditional operation), and so on.

**Fig. 2:**
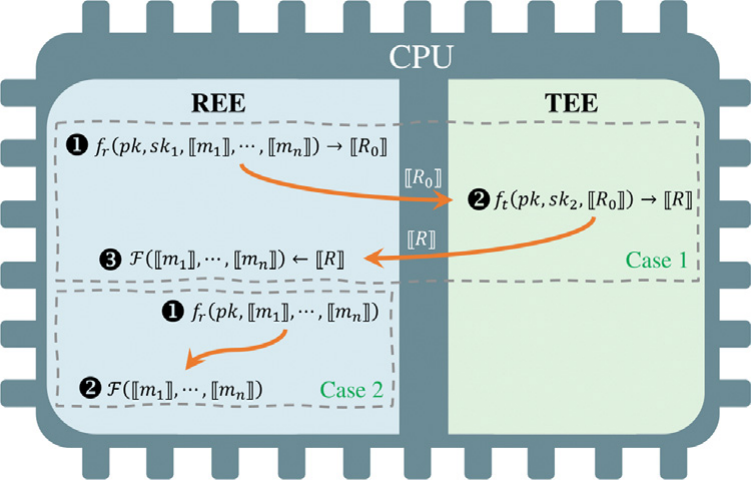
**Typical workflow of TRUST**.

Fig. 2 shows a typical workflow of TRUST. As depicted in Fig. 2, TRUST reconstructs the computation $F(⟦m_{1}⟧,\ldots ,⟦m_{n}⟧)$ into $f_{r}\left(pk,sk_{1},⟦m_{1}⟧,\ldots ,⟦m_{n}⟧\right)\to ⟦R_{0}⟧$ and $f_{t}\left(pk,sk_{2},⟦R_{0}⟧\right)\to F\left(⟦m_{1}⟧,\ldots ,⟦m_{n}⟧\right)$ (Case 1), or $f_{r}\left(pk,⟦m_{1}⟧,\ldots ,⟦m_{n}⟧\right)$ (Case 2), where $⟦R_{0}⟧$ is an intermediate result, and n means the amount of operands.

### 5.2 Protocols design

In this section, we detail operations supported by TRUST, including multiplication, comparison, equality, absolute value, and ternary conditional operation. Obviously, TRUST is easy to achieve addition and scalar multiplication, as they are homomorphic features of FastPaiTD. Taking addition as an example, $F(⟦m_{1}⟧,⟦m_{2}⟧)$ is formulated as $F\left(⟦m_{1}⟧,⟦m_{2}⟧\right)\to ⟦m_{1}+m_{2}⟧$. In this case, $f_{r}$ is formulated as $f_{r}\left(⟦m_{1}⟧,⟦m_{2}⟧\right)=⟦m_{1}⟧\cdot ⟦m_{2}⟧\text{mod}N^{2}$, while $f_{t}$ can be omitted. Following the previous work [7], we assume $m_{1},m_{2},\ldots ,m_{n}\in \left(-2^{\ell },2^{\ell }\right)$, where ℓ is a constant (e.g., ℓ=32).

**Algorithm 1:** $F_{\mathrm{mul}}\left(⟦m_{1}⟧,⟦m_{2}⟧\right)\to ⟦m_{1}\cdot m_{2}⟧$



Algorithm 1 shows a secure multiplication protocol following Definition 1. Formally, $F(⟦m_{1}⟧,\ldots ,⟦m_{n}⟧)$ is denoted by $F_{\mathrm{mul}}\left(⟦m_{1}⟧,⟦m_{2}⟧\right)\to ⟦m_{1}\cdot m_{2}⟧$, where n=2. At Step 1, REE takes as input keys (pk and $sk_{1}$), $⟦m_{1}⟧$, and $⟦m_{2}⟧$, and outputs $⟦m_{1}+r⟧$, ${\left[m_{1}+r\right]}_{1}$, $⟦m_{2}⟧$, and $⟦-r\cdot m_{2}⟧$, where r is a σ-bit (e.g., σ=128) random number, and ←$ means random sampling in this work.

After receiving the output of REE, TEE first obtains $m_{1}+r$ by calling PDec and TDec sequentially and then outputs the final result ⟦R⟧. According to the homomorphic properties of FastPaiTD, we have

(6) $$⟦m_{2}{⟧}^{m_{1}+r}\cdot ⟦-r\cdot m_{2}⟧\cdot \mathrm{Enc}\left(pk,0\right)\to ⟦m_{1}\cdot m_{2}+r\cdot m_{2}-r\cdot m_{2}+0⟧.$$

Thus, it is easy to verify $R=m_{1}\cdot m_{2}$. In other words, $F_{\mathrm{mul}}$ outputs $⟦m_{1}\cdot m_{2}⟧$ correctly.

Algorithm 2 depicts a secure comparison protocol following Definition 1. Formally, $F(⟦m_{1}⟧,\ldots ,⟦m_{n}⟧)$ is denoted by $F_{\mathrm{cmp}}\left(⟦m_{1}⟧,⟦m_{2}⟧\right)\to ⟦\mu ⟧$, where μ=0 when $m_{1}\geq m_{2}$, otherwise ($m_{1}<m_{2}$), μ=1. At Step 1, REE takes as input keys (pk and $sk_{1}$), $⟦m_{1}⟧$, and $⟦m_{2}⟧$, and outputs ⟦d⟧, ${\left[d\right]}_{1}$, and ⟦π⟧. Specifically, REE first generates $r_{1}$ and $r_{2}$ ($r_{1}+r_{2}>\frac{N}{2}$) by random sampling. After that, REE randomly samples π from {0,1} and computes ⟦d⟧. According to the homomorphic properties of FastPaiTD, we have

(7) $$d=\left\{ \begin{array}{cc} r_{1}\cdot \left(m_{1}-m_{2}+1\right)+r_{2}, & \pi =0;\\ r_{1}\cdot \left(m_{2}-m_{1}\right)+r_{2}. & \pi =1; \end{array}\right.$$

After receiving the output of REE, TEE first obtains d by calling PDec and TDec sequentially. Then, TEE computes and returns ⟦R⟧ to REE. It is easy to verify

(8) $$R=\mu_{0}+\left(1-2\mu_{0}\right)\cdot \pi .$$

If $\mu_{0}=0$ (i.e., $d>\frac{N}{2}$), we have R=π, whereas $\mu_{0}=1$ (i.e., $d<\frac{N}{2}$), we have R=1−π. As shown in Eq. (8), the value of R depends on the variables $\mu_{0}$ and π, while the value of $\mu_{0}$ depends on d.

**Algorithm 2:** $F_{\mathrm{cmp}}\left(⟦m_{1}⟧,⟦m_{2}⟧\right)\to ⟦\mu ⟧$



*Case 1* (π=0): If $r_{1}\cdot \left(m_{1}-m_{2}+1\right)+r_{2}>\frac{N}{2}$ (i.e., $d>\frac{N}{2}$), we have $(m_{1}-m_{2}+1)\geq 1$ because $r_{1}+r_{2}>\frac{N}{2}$ and $r_{2}<\frac{N}{2}$. In this case, $m_{1}\geq m_{2}$, while $R=\mu_{0}=0$. If $r_{1}\cdot \left(m_{1}-m_{2}+1\right)+r_{2}<\frac{N}{2}$ (i.e., $d<\frac{N}{2}$), we have $(m_{1}-m_{2}+1)\leq 0$ because $r_{1}+r_{2}>\frac{N}{2}$ and $r_{2}<\frac{N}{2}$. In this case, $m_{1}<m_{2}$, while $R=\mu_{0}=1$. In other words, when π=0, if $m_{1}\geq m_{2}$, $F_{\mathrm{cmp}}$ outputs μ=0, otherwise ($m_{1}<m_{2}$), outputs μ=1.

*Case 2* (π=1): If $r_{1}\cdot \left(m_{2}-m_{1}\right)+r_{2}>\frac{N}{2}$ (i.e., $d>\frac{N}{2}$), we have $m_{2}-m_{1}\geq 1$ because $r_{1}+r_{2}>\frac{N}{2}$ and $r_{2}<\frac{N}{2}$. In this case, $m_{1}<m_{2}$, while $R=1-\mu_{0}=1$. If $r_{1}\cdot \left(m_{2}-m_{1}\right)+r_{2}<\frac{N}{2}$ (i.e., $d<\frac{N}{2}$), we have $m_{2}-m_{1}\leq 0$ because $r_{1}+r_{2}>\frac{N}{2}$ and $r_{2}<\frac{N}{2}$. In this case, $m_{1}\geq m_{2}$, while $R=1-\mu_{0}=0$. In other words, when π=1, if $m_{1}\geq m_{2}$, $F_{\mathrm{cmp}}$ outputs μ=0, otherwise ($m_{1}<m_{2}$), outputs μ=1.

Taken together, either π=0 or π=1, $F_{\mathrm{cmp}}$ always computes ⟦μ⟧ correctly.

**Algorithm 3:** $F_{\mathrm{eql}}\left(⟦m_{1}⟧,⟦m_{2}⟧\right)\to ⟦\mu ⟧$



Algorithms 1 and 2 denote a linear secure operation and a non-linear secure operation, respectively. In addition to the non-linear comparison operation, an equality operation is also common. Algorithm 3 lists the details of a secure equality protocol. Formally, $F(⟦m_{1}⟧,\ldots ,⟦m_{n}⟧)$ is denoted by $F_{\mathrm{eql}}\left(⟦m_{1}⟧,⟦m_{2}⟧\right)\to ⟦\mu ⟧$, where μ=0 if $m_{1}=m_{2}$ holds, otherwise ($m_{1}\neq m_{2}$), μ=1. $F_{\mathrm{eql}}$ is concreted to two critical steps. At Step 1, REE computes $⟦d_{1}⟧$ and $⟦d_{2}⟧$ in the same way as ⟦d⟧ in Algorithm 2, and outputs $⟦d_{1}⟧$, ${\left[d_{1}\right]}_{1}$, $⟦\pi_{1}⟧$, $⟦d_{2}⟧$, ${\left[d_{2}\right]}_{1}$, and $⟦\pi_{2}⟧$. According to the homomorphic properties of FastPaiTD, we have

(9) $$d_{1}=\left\{ \begin{array}{cc} r_{1}\cdot \left(m_{1}-m_{2}+1\right)+r_{2}, & \pi_{1}=0;\\ r_{1}\cdot \left(m_{2}-m_{1}\right)+r_{2}, & \pi_{1}=1; \end{array}\right.$$

(10) $$d_{2}=\left\{ \begin{array}{cc} r_{1}^{\prime }\cdot \left(m_{2}-m_{1}+1\right)+r_{2}^{\prime }, & \pi_{2}=0;\\ r_{1}^{\prime }\cdot \left(m_{1}-m_{2}\right)+r_{2}^{\prime }. & \pi_{2}=1; \end{array}\right.$$

At Step 2, TEE obtains $d_{1}$ and $d_{2}$ by calling PDec and TDec sequentially. Essentially, $d_{1}$ and $d_{2}$ imply the magnitude relationship between $m_{1}$ and $m_{2}$, and the relationship between $m_{2}$ and $m_{1}$, respectively. Mathematically, $m_{1}=m_{2}$ is equivalent to $m_{1}\geq m_{2}\&m_{2}\geq m_{1}$. Inspired by this idea, at Step 2, TEE computes and returns ⟦R⟧ to REE, where

(11) $$R=\mu_{0}+\left(1-2\mu_{0}\right)\cdot \pi_{1}+\mu_{0}^{\prime }+\left(1-2\mu_{0}^{\prime }\right)\cdot \pi_{2}.$$

**Algorithm 4:** $F_{\mathrm{abs}}\left(⟦m_{1}⟧\right)\to ⟦\left|m_{1}\right|⟧$



According to Algorithm 2, if $m_{1}\geq m_{2}$, $\mu_{0}+\left(1-2\mu_{0}\right)\cdot \pi_{1}=0$. Additionally, if $m_{2}\geq m_{1}$, $\mu_{0}^{\prime }+\left(1-2\mu_{0}^{\prime }\right)\cdot \pi_{2}=0$. And whether it is $\mu_{0}+\left(1-2\mu_{0}\right)\cdot \pi_{1}$ or $\mu_{0}^{\prime }+\left(1-2\mu_{0}^{\prime }\right)\cdot \pi_{2}$, the result can only be 0 or 1. Furthermore, $\mu_{0}+\left(1-2\mu_{0}\right)\cdot \pi_{1}$ and $\mu_{0}^{\prime }+\left(1-2\mu_{0}^{\prime }\right)\cdot \pi_{2}$ cannot both be equal to 1 as $m_{1}<m_{2}\&m_{2}<m_{1}$ cannot be true at the same time. Therefore, R=0 if and only if $m_{1}\geq m_{2}\&m_{2}\geq m_{1}$ (i.e., $m_{1}=m_{2}$). Also, R=1 if and only if $m_{1}>m_{2}\&m_{2}<m_{1}$ or $m_{1}<m_{2}\&m_{2}>m_{1}$. In other words, if $m_{1}=m_{2}$, $F_{\mathrm{eql}}$ outputs ⟦0⟧, otherwise $m_{1}\neq m_{2}$ (i.e., $m_{1}>m_{2}$ or $m_{2}>m_{1}$), it outputs ⟦1⟧. Thus, we say that $F_{\mathrm{eql}}$ outputs ⟦μ⟧ correctly.

Now, we discuss one unary operation (i.e., absolute value). If m≥0, |m|=m, otherwise (m<0), |m|=−m. Thus, |m| can be denoted by |m|=(1−2μ)·m, where μ=0 when m≥0, otherwise (m<0), μ=1.

Inspired by the above observation, Algorithm 4 depicts a secure absolute value protocol. Formally, $F(⟦m_{1}⟧)$ is denoted by $F_{\mathrm{abs}}\left(⟦m_{1}⟧\right)\to ⟦\left|m\right|⟧$, where n is set as n=1. Specifically, at Step 1, REE takes as input keys and $⟦m_{1}⟧$, and outputs four ciphertexts including ⟦d⟧, ${\left[d\right]}_{1}$, $⟦m_{1}⟧$, and $⟦\pi \cdot m_{1}⟧$, where $⟦\pi \cdot m_{1}⟧\leftarrow ⟦m_{1}{⟧}^{\pi }\cdot \mathrm{Enc}\left(pk,0\right)$. According to the homomorphic properties of FastPaiTD, we have

(12) $$d=\left\{ \begin{array}{cc} r_{1}\cdot \left(m_{1}+1\right)+r_{2}, & \pi =0;\\ r_{1}\cdot \left(-m_{1}\right)+r_{2}. & \pi =1; \end{array}\right.$$

At Step 2, TEE obtains d by calling PDec and TDec sequentially. From Eq. (12), we see that d essentially implies the magnitude relationship between $m_{1}$ and 0. After that, TEE computes and returns ⟦R⟧ to REE. It is easy to verify

(13) $$R=\left(1-2\mu_{0}\right)\cdot m_{1}+\left(4\mu_{0}-2\right)\cdot \pi \cdot m_{1},$$

where $\mu_{0}=0$ when $d>\frac{N}{2}$, otherwise, $\mu_{0}=1$.

As $|m_{1}|=\left(1-2\mu \right)\cdot m_{1}$, where μ=0 when $m_{1}\geq 0$, otherwise, μ=1, according to Algorithm 2, we see $\mu =\mu_{0}+\left(1-2\mu_{0}\right)\cdot \pi$. Then, we have

(14) $$\begin{array}{cc} |m_{1}| & =\left[1-2\cdot \left(\mu_{0}+\left(1-2\mu_{0}\right)\cdot \pi \right)\right]\cdot m_{1}\\ & =\left(1-2\mu_{0}\right)\cdot m_{1}+\left(4\mu_{0}-2\right)\cdot \pi \cdot m_{1}\\ & =R. \end{array}$$

Thus, we can say that $F_{\mathrm{abs}}$ oputputs $⟦|m_{1}|⟧$ correctly.

Finally, we describe a secure ternary (conditional) operation. Formally, the ternary conditional operation can be formulated as “*if a then b else c*”. Without loss of generality, $F(⟦m_{1}⟧,⟦m_{2}⟧,⟦m_{3}⟧)$ is denoted by $F_{\mathrm{trn}}\left(⟦m_{1}⟧,⟦m_{2}⟧,⟦m_{3}⟧\right)\to ⟦m⟧$, where if $m_{1}=1$, then $m=m_{2}$, else $m=m_{3}$. Note that $m=m_{2}+\mu \cdot \left(m_{3}-m_{2}\right)$ is always true if μ=0 when $m_{1}=1$, otherwise ($m_{1}\neq 1$), μ=1.

Algorithm 5 shows the details of the secure ternary protocol. Specifically, at Step 1, REE computes $⟦d_{1}⟧$ and $⟦d_{2}⟧$ in the same way as those in Algorithm 3, and outputs eight ciphertexts, where $⟦\pi_{1}\cdot \left(m_{3}-m_{2}\right)⟧\leftarrow ⟦m_{3}-m_{2}{⟧}^{\pi_{1}}\cdot \mathrm{Enc}\left(pk,0\right)$, and $⟦\pi_{2}\cdot \left(m_{3}-m_{2}\right)⟧\leftarrow ⟦m_{3}-m_{2}{⟧}^{\pi_{2}}\cdot \mathrm{Enc}\left(pk,0\right)$. According to the homomorphic properties of FastPaiTD, we have

(15) $$d_{1}=\left\{ \begin{array}{cc} r_{1}\cdot m_{1}+r_{2}, & \pi_{1}=0;\\ r_{1}\cdot \left(1-m_{1}\right)+r_{2}, & \pi_{1}=1; \end{array}\right.$$

(16) $$d_{2}=\left\{ \begin{array}{cc} r_{1}^{\prime }\cdot \left(2-m_{1}\right)+r_{2}^{\prime }, & \pi_{2}=0;\\ r_{1}^{\prime }\cdot \left(m_{1}-1\right)+r_{2}^{\prime }. & \pi_{2}=1; \end{array}\right.$$

At Step 2, TEE first obtains $d_{1}$ and $d_{2}$ by calling PDec and TDec sequentially. In fact, $d_{1}$ and $d_{2}$ imply the magnitude relationship between $m_{1}$ and 1, and the relationship between 1 and $m_{1}$, respectively. After that, TEE computes and returns ⟦R⟧ to REE. It is easy to verify

(17) $$\begin{array}{cc} R= & \;m_{2}+\left(\mu_{0}+\mu_{0}^{\prime }\right)\cdot \left(m_{3}-m_{2}\right)+\left(1\;-\;2\mu_{0}\right)\;\cdot \;\pi_{1}\;\cdot \;\left(m_{3}-m_{2}\right)\\ & +\left(1-2\mu_{0}^{\prime }\right)\;\cdot \;\pi_{2}\;\cdot \;\left(m_{3}-m_{2}\right). \end{array}$$

As $m=m_{2}+\mu \cdot \left(m_{3}-m_{2}\right)$, where μ=0 when $m_{1}=1$, otherwise, μ=1. According to Eq. (11), $\mu =\mu_{0}+\left(1-2\mu_{0}\right)\cdot \pi_{1}+\mu_{0}^{\prime }+\left(1-2\mu_{0}^{\prime }\right)\cdot \pi_{2}$ holds. Then, we have

(18) $$\begin{array}{cc} m= & \;m_{2}+\left[\mu_{0}+\left(1-2\mu_{0}\right)\cdot \pi_{1}+\mu_{0}^{\prime }+\left(1-2\mu_{0}^{\prime }\right)\cdot \pi_{2}\right]\\ & \cdot (m_{3}-m_{2})\\ = & \;m_{2}+\left(\mu_{0}+\mu_{0}^{\prime }\right)\cdot \left(m_{3}-m_{2}\right)\;+\;\left(1\;-\;2\mu_{0}\right)\;\cdot \;\pi_{1}\;\cdot \;\left(m_{3}-m_{2}\right)\\ & +\left(1-2\mu_{0}^{\prime }\right)\cdot \pi_{2}\cdot \left(m_{3}-m_{2}\right)\\ = & \;R. \end{array}$$

Thus, we say $F_{\mathrm{trn}}$ outputs ⟦m⟧ correctly.

**Algorithm 5:** $F_{\mathrm{trn}}\left(⟦m_{1}⟧,⟦m_{2}⟧,⟦m_{3}⟧\right)\to ⟦m⟧$



## 6 Seat details

In this section, we describe SEAT, a secure data trading solution based on our proposed TRUST. Data trading is the key to driving the digital economy [52], [53]. SEAT aims to answer the following two questions faced by existing data trading:

*(1) Can the trading data be prevented from being copied?*

*(2) If the answer to the first question is no, can the trading data be prevented from data resale?*

Our proposed SEAT argues that SOC is one of the feasible methods to prevent data resale. Roughly speaking, SEAT trades data use right instead of data ownership through SOC.

**Fig. 3:**
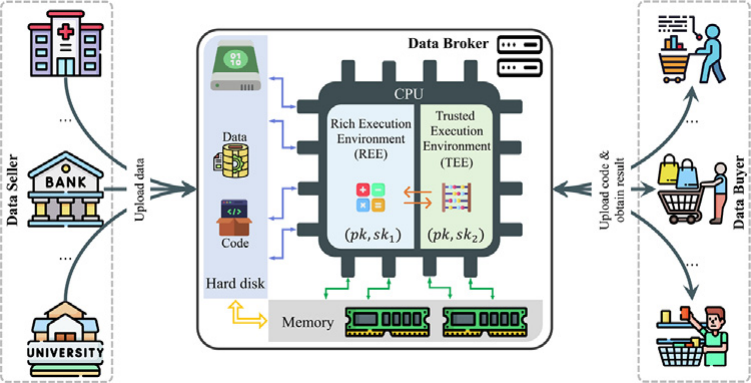
**System architecture of SEAT**.

Fig. 3 shows the system architecture of SEAT that consists of three entities: data seller, data broker, and data buyer. The data seller owning data sells the data use right to the data buyer with the help of the data broker. The workflow of SEAT involves following steps.

❶ The data seller initializes system keys including a public/private key pair (pk,sk) and two pairs of threshold keys $\left(sk_{1},sk_{2}\right)$ and $\left({\text{sk}}_{1},{\text{sk}}_{2}\right)$. After that, the data seller encrypts data item by item through calling Enc, and transmits ciphertexts and corresponding keys to the data broker.

❷ The data buyer submits data usage requirements to the data broker.

❸ The data broker performs computations as required by the data buyer through calling SOC operations of TRUST, and then returns the encrypted result ⟦R⟧ to the data seller.

❹ The data seller calculates $\mathrm{PDec}\left({\text{sk}}_{1},⟦R⟧\right)\to {\left[R\right]}_{1}$ and sends $\langle ⟦R⟧,{\left[R\right]}_{1},{\text{sk}}_{2}\rangle$ to the data buyer.

❺ The data buyer calls $\mathrm{PDec}\left({\text{sk}}_{2},⟦R⟧\right)\to {\left[R\right]}_{2}$ and $\mathrm{TDec}({\left[R\right]}_{1},{\left[R\right]}_{2})$ to obtain the final result R.

From the workflow of SEAT, we see that either the data broker or the data buyer fails to learn the origin data of the data seller. In other words, no one of the data broker and the data buyer can resell the origin data from the data seller. Although the data broker can copy the encrypted origin data, he is only able to perform operations over ciphertexts and lacks decryption capabilities, thereby being incapable to provide services requiring access to plaintext data, though limited ciphertext operations may still be feasible.

## 7 Security analysis

In this section, we aim to demonstrate that TRUST does not leak any outsourced data to REE and TEE under the assumption of our threat model.

Lemma 1 Dec(sk,⟦m⟧·Enc(pk,0))=Dec(sk,⟦m⟧) *is always true, and* ⟦m⟧·Enc(pk,0)≠⟦m⟧ *is also true.*

Proof We first prove that Dec(sk,⟦m⟧·Enc(pk,0))=Dec(sk,⟦m⟧) is true. According to the additive homomorphism of FastPaiTD, we have

(19) ⟦m⟧·Enc(pk,0)=⟦m+0⟧.

And since m+0=m is always true $\forall m\in Z_{N}$, Dec(sk,⟦m⟧·Enc(pk,0))=m. Thus, Dec(sk,⟦m⟧·Enc(pk,0))=Dec(sk,⟦m⟧) is always true.

According to Eq. (1), we have

(20) $$⟦m⟧\cdot \mathrm{Enc}(pk,0)=⟦m⟧\cdot {\left(1+N\right)}^{0}\cdot {\left(h^{r}\;\text{mod}\;N\right)}^{N}=⟦m⟧\cdot {\left(h^{r}\;\text{mod}\;N\right)}^{N},$$

where $r\leftarrow \${\left\{0,1\right\}}^{4\kappa }$. Obviously, ${\left(h^{r}\;\;\;\;\text{mod}N\right)}^{N}\neq 1$, so ⟦m⟧·Enc(pk,0)≠⟦m⟧. □

Lemma 2 *When* $m\in (-2^{\ell },2^{\ell })$*,* $r\leftarrow \${\left\{0,1\right\}}^{\sigma }$*, and* $2^{\sigma -\ell +2}$ *is a negligible function,* m+r *is a chosen-plaintext attack secure one-time key encryption scheme, where* m *and* r *are the plaintext to be encrypted and the key, respectively.*

Proof Refer to the work [7] for the details of the proof. □

Theorem 1 *Given* $\left(⟦m_{1}⟧,⟦m_{2}⟧\right)$*,* $F_{\mathrm{mul}}$ *securely computes* $⟦m_{1}\cdot m_{2}⟧$*, and does not leak* $m_{1}$*,* $m_{2}$*, or* $m_{1}\cdot m_{2}$ *to REE and TEE.*

Proof At Step 1, REE only performs operations over $⟦m_{1}⟧$ and $⟦m_{2}⟧$. As long as FastPaiTD is secure, none of $m_{1}$, $m_{2}$, or $m_{1}\cdot m_{2}$ are leaked to REE.

At Step 2, although TEE can learn $m_{1}+r$, he fails to obtain $m_{1}$ according to Lemma 2. Thus, we say that $m_{1}$ is not leaked to TEE. And since FastPaiTD is secure, TEE cannot obtain $m_{2}$ and $m_{1}\cdot m_{2}$.

At Step 3, REE can learn $⟦m_{2}{⟧}^{m_{1}+r}\cdot ⟦-r\cdot m_{2}⟧\cdot \mathrm{Enc}\left(pk,0\right)$. Given $⟦m_{2}{⟧}^{m_{1}+r}\cdot ⟦-r\cdot m_{2}⟧$, REE is easy to learn $m_{1}$ under knowing $⟦m_{2}⟧$, r, and $⟦-r\cdot m_{2}⟧$. According to the computational indistinguishability experiment [7], REE successfully wins the experiment. Specifically, REE randomly chooses $m_{0}$ and $m_{1}$ and sends them to a challenger. After that, the challenger b←${0,1} and computes and returns $⟦m_{2}{⟧}^{m_{b}+r}\cdot ⟦-r\cdot m_{2}⟧$ to REE. In this case, if

$$⟦m_{2}{⟧}^{m_{b}+r}\cdot ⟦-r\cdot m_{2}⟧=⟦m_{2}{⟧}^{m_{0}+r}\cdot ⟦-r\cdot m_{2}⟧,$$

REE ouputs b=0, while

$$⟦m_{2}{⟧}^{m_{b}+r}\cdot ⟦-r\cdot m_{2}⟧=⟦m_{2}{⟧}^{m_{1}+r}\cdot ⟦-r\cdot m_{2}⟧,$$

REE ouputs b=1. In other words, REE always successfully guesses b and wins the experiment.

However, as ⟦m⟧·Enc(pk,0)≠⟦m⟧ (see Lemma 2), $⟦m_{2}{⟧}^{m_{b}+r}\cdot ⟦-r\cdot m_{2}⟧\cdot \mathrm{Enc}\left(pk,0\right)$ may be equal to $⟦m_{2}{⟧}^{m_{0}+r}\cdot ⟦-r\cdot m_{2}⟧\cdot \mathrm{Enc}\left(pk,0\right)$ or $⟦m_{2}{⟧}^{m_{1}+r}\cdot ⟦-r\cdot m_{2}⟧\cdot \mathrm{Enc}\left(pk,0\right)$. Thus, REE fails to guess b with a probability greater than 1/2. In other words, REE cannot win the experiment, so he fails to learn $m_{1}$. And since FastPaiTD is secure, $m_{2}$ and $m_{1}\cdot m_{2}$ are not leaked to REE. □

Theorem 2 *Given* $\left(⟦m_{1}⟧,⟦m_{2}⟧\right)$*,* $F_{\mathrm{cmp}}$ *securely compares* $⟦m_{1}⟧$ *and* $⟦m_{2}⟧$*, and does not leak* $m_{1}$*,* $m_{2}$*, and* μ *to REE and TEE.*

Proof At Step 1, REE only performs operations over $⟦m_{1}⟧$ and $⟦m_{2}⟧$. As long as FastPaiTD is secure, $m_{1}$, $m_{2}$, and μ are leaked to REE.

At Step 2, TEE can learn d and $\mu_{0}$, but it is easy to demonstrate $\mu_{0}$ and d do not leak $m_{1}$, $m_{2}$, and μ. Specifically, according to Algorithm 2, we have $\mu =\mu_{0}+\left(1-2\mu_{0}\right)\cdot \pi$. As FastPaiTD is secure, TEE fails to learn π under knowing ⟦π⟧. Thus, given $\mu_{0}$ and ⟦π⟧, μ is not leaked to TEE.

Now, we adopt the computational indistinguishability experiment to prove that d does not disclose $m_{1}$ and $m_{2}$. Let TEE act as an adversary. Then, TEE randomly generates $\left(m_{1,0},m_{2,0}\right)$ and $\left(m_{1,1},m_{2,1}\right)$ and sends them to a challenger. Mathematically, $\left(m_{1,0}=m_{2,0}\right)$ and $\left(m_{1,1}=-2^{\ell }+1,m_{2,1}=2^{\ell }-1\right)$, TEE obtains the highest probability of guessing success. After that, the challenger generates b,π←${0,1} and $r_{1}\leftarrow \${\left\{0,1\right\}}^{\sigma },r_{2}\leftarrow \$\left(\frac{N}{2}-r_{1},\frac{N}{2}\right)$, and computes

(21) $$\begin{array}{c} d=\left\{ \begin{array}{cc} r_{1}+r_{2}, & \;\text{for}\;b=0,\pi =0\\ r_{1}\cdot 0+r_{2}, & \;\text{for}\;b=0,\pi =1\\ r_{1}\cdot \left(-2^{\ell +1}+1\right)+r_{2}, & \;\text{for}\;b=1,\pi =0\\ r_{1}\cdot \left(2^{\ell +1}-2\right)+r_{2}. & \;\text{for}\;b=1,\pi =1 \end{array}\right. \end{array}$$

The challenger returns d to TEE. Subsequently, TEE guesses $b^{\prime }=0$ or $b^{\prime }=1$. In this case, the probability of guessing success of TEE can be formulated as

(22) $$\mathrm{Pr}\left[b^{\prime }=b|d\right]\leq \frac{1}{2}+\frac{1}{2}\cdot \frac{1-(2^{\ell +1}+1)}{2^{\sigma }}=\frac{1}{2}+\frac{2^{\ell }}{2^{\sigma }}.$$

As $2^{\ell }\cdot 2^{-\sigma }$ is a negligible function [7], $\mathrm{Pr}\left[b^{\prime }=b|d\right]\leq \frac{1}{2}+\text{negl}\left[\sigma \right]$ holds. Thus, we say that the probability of guessing success of TEE is negligible. In other words, TEE fails to learn $m_{1}$ and $m_{2}$ from d.

At Step 3, REE only obtains an encrypted ⟦μ⟧, so μ is not disclosed to REE. □

Theorem 3 *Given* $\left(⟦m_{1}⟧,⟦m_{2}⟧\right)$*,* $F_{\mathrm{eql}}$ *securely determines the relationship between* $⟦m_{1}⟧$ *and* $⟦m_{2}⟧$*, and does not leak* $m_{1}$*,* $m_{2}$*, and* μ *to REE and TEE.*

Proof At Steps 1 and 3, REE either performs operations over $⟦m_{1}⟧$ and $⟦m_{2}⟧$ or obtains an encrypted ⟦μ⟧, therefore, $m_{1}$, $m_{2}$, and μ are not leaked to REE.

At Step 2, although TEE can learn $d_{1}$ and $d_{2}$, according to Theorem 2, no $m_{1}$ or $m_{2}$ is disclosed to TEE. From Algorithm 3, we have $\mu =\mu_{0}+\left(1-2\mu_{0}\right)\cdot \pi_{1}+\mu_{0}^{\prime }+\left(1-2\mu_{0}^{\prime }\right)\cdot \pi_{2}$. Thus, TEE cannot obtain μ as $\pi_{1}$ and $\pi_{2}$ is unknown. □

Theorem 4 *Given* $⟦m_{1}⟧$*,* $F_{\mathrm{abs}}$ *securely computes* $⟦|m_{1}|⟧$*, and does not leak* $|m_{1}|$ *and* $m_{1}$ *to REE and TEE.*

Proof At Step 1, REE performs operations over $⟦m_{1}⟧$, thus, $m_{1}$ is not leaked to REE when FastPaiTD is secure.

At Step 2, according to Lemma 2 and Theorem 2, TEE fails to learn π and $m_{1}$. And since $|m_{1}|=\left(1-2\mu_{0}\right)\cdot m_{1}+\left(4\mu_{0}-2\right)\cdot \pi \cdot m_{1}$, $|m_{1}|$ is not disclosed to TEE due to neither π nor $m_{1}$ being leaked.

At Step 3, REE only learns $⟦|m_{1}|⟧$, thus, as long as FastPaiTD is secure and Lemma 2 holds, REE fails to obtain $|m_{1}|$. □

Theorem 5 *Given* $\left(⟦m_{1}⟧,⟦m_{2}⟧,⟦m_{3}⟧\right)$*,* $F_{\mathrm{trn}}$ *securely computes* ⟦m⟧*, and does not leak* $m_{1},m_{2},m_{3}$ *and* m *to REE and TEE.*

Proof At Step 1, REE only performs operations over $⟦m_{1}⟧$, $⟦m_{2}⟧$ and $⟦m_{3}⟧$, therefore, no $m_{1},m_{2}$, or $m_{3}$ are leaked to REE as long as FastPaiTD remains secure.

At Step 2, according to Lemma 2 and Theorem 2, TEE fails to learn $\pi_{1},\pi_{2}$ and $m_{1}$. And since $m=m_{2}+\left[\left(\mu_{0}+\mu_{0}^{\prime }\right)+\left(1-2\mu_{0}\right)\cdot \pi_{1}+\left(1-2\mu_{0}^{\prime }\right)\cdot \pi_{2}\right]\cdot \left(m_{3}-m_{2}\right)$, m is not leaked to TEE. Additionally, as long as FastPaiTD is secure, given $⟦m_{2}⟧$ and $⟦m_{3}-m_{2}⟧$, no $m_{2}$ or $m_{3}$ is disclosed to TEE.

At Step 3, REE only obtains ⟦m⟧, thus, m is not leaked to REE under FastPaiTD being secure and Lemma 2 holding. □

## 8 Experimental evaluations

In this section, we thoroughly evaluate the proposed TRUST and SEAT based on TRUST. Note that all experiments are measured 1000 times and take their average as the experimental result.

### 8.1 Experimental setup

**System Configuration**. To evaluate the performance of TRUST, we construct the framework using Intel® SGX in hardware mode as a specific implementation of TEE. In detail, we implement TRUST^3^ and SEAT in C++ and conduct experiments on a single server. The server runs a 64-bit Ubuntu 20.04 LTS operating system and is equipped with an Intel® Xeon® Silver 4410Y CPU @ 2.00 GHz, along with 128GB of memory, 64GB of which is allocated to the Intel® SGX’s EPC.

**Network Configuration**. Adopting the twin-server architecture perspective, network communications specifically refers to inter-server communication (i.e., network interactions between servers). To ensure a fair performance comparison, the schemes (i.e., SOCI^+^ [13], SOCI [7], and POCF [23]) based on a twin-server architecture are simulated using two isolated processes on the same physical server. Each process represents a different entity, such as a cloud platform or a computation service provider. All experiments are conducted in a LAN environment with a bandwidth of 46.4 Gbps (5.8 GB/s). In contrast, we simulate data transmission between trusted and untrusted environments by implementing secure interfaces (e.g., ECall and OCall functions provided by enclave). In our testbed, communication costs are defined as the volume of data transmitted between processes.

Note that TRUST also adopts the offline-online mechanism proposed by Zhao *et al* [13]. to accelerate the computations. Roughly speaking, in this work, REE and TEE environments independently generate tuples (i.e., a set contains random numbers, encrypted random numbers, or ciphertexts of 0 and 1) during the offline phase. Specifically, the encryptions of random numbers (e.g., $r_{i}$ and $r_{i}^{\prime }$, where i∈{1,2}) and certain constants (e.g., ⟦0⟧ or ⟦1⟧) are precomputed offline, while the online phase handles only the remaining operations from the offline phase. For further details, refer to the work [13].

### 8.2 Basic cryptographic primitive operations evaluations

This subsection benchmarks the performance of TRUST’s basic cryptographic primitives against representative schemes including SOCI^+^, SOCI and POCF, focusing on runtime and storage costs. Specifically, we set the bit length of the modulus N to 2048 bits to achieve 112-bit security.

From Table 1, we see that TRUST provides extremely fast encryption and decryption speeds, with execution times of 0.346 ms for Enc, 1.617 ms for Dec, and 10.104 ms for a complete threshold decryption (i.e., PDec+TDec). TRUST outperforms SOCI and POCF in terms of runtime and storage costs, and is comparable to SOCI^+^ only a minor difference (+ 0.7 ms) in PDec+TDec. This overhead, clearly, is not significant. We believe the marginal cost is a worthwhile trade-off, considering the architectural benefit of a single-server model. Thus, TRUST demonstrates the best performance in basic cryptographic primitive operations compared to existing solutions.

**Table 1:** **Comparison of Runtime and Storage Costs for Basic Cryptographic Primitive Operations (112-bit Security Level)**.

Schemes	KGen	Enc	Dec	PDec+TDec^a^	Addition	Scalar-Multiplication	Subtraction	pk	sk	ciphertext
TRUST	**61.779 ms**	**0.346 ms**	**1.617 ms**	10.104 ms	**0.003 ms**	**0.021 ms**	**0.035 ms**	**0.500 KB**	**0.055 KB**	**0.500 KB**
SOCI^+^[13]	62.334 ms	0.347 ms	1.619 ms	**9.401 ms**	0.003 ms	0.021 ms	0.035 ms	0.500 KB	0.055 KB	0.500 KB
SOCI [7]	75.776 ms	7.076 ms	7.072 ms	15.005 ms	0.003 ms	0.021 ms	0.035 ms	0.500 KB	0.250 KB	0.500 KB
POCF [23]	74.978 ms	7.053 ms	7.041 ms	14.928 ms	0.003 ms	0.021 ms	0.035 ms	0.500 KB	0.250 KB	0.500 KB

a PDec+TDec refers to executing PDec twice (with keys $sk_{1}$ and $sk_{2}$, respectively) followed by one TDec operation.

### 8.3 Protocol evaluations

Table 2 presents the computation and communication costs of TRUST, SOCI^+^, SOCI, and POCF at the 112-bit security level. The experimental results show that TRUST outperforms SOCI^+^, SOCI, and POCF in terms of both computation and communication costs. Specifically, TRUST reduces computation costs by 1.6 - 2.1 times compared to SOCI^+^, which is the state-of-the-art solution for secure multiplication and secure comparison within the FastPaiTD-based twin-server architecture. One possible explanation is that TRUST designs a more efficient multiplication protocol and presents a more concise system architecture.

**Table 2:** **Comparison of Computation and Communication Costs (112-bit Security Level)**.

Protocols	Computation costs (ms)				Communication costs (KB)			
	TRUST	SOCI^+^[13]	SOCI [7]	POCF [23]	TRUST	SOCI^+^[13]	SOCI [7]	POCF [23]
$F_{\mathrm{mul}}$	**11.532**	23.084	62.120	71.271	**0**	1.499	2.499	2.499
$F_{\mathrm{cmp}}$	**11.476**	24.941	46.801	47.791	**0**	1.499	1.499	1.499
$F_{\mathrm{eql}}$	**23.013**	36.224	85.881	253.658	**0**	3.497	3.497	7.998
$F_{\mathrm{abs}}$	**11.024**	20.965	49.115	48.728	**0**	2.498	2.498	2.498
$F_{\mathrm{trn}}$	**22.004**	36.228	70.913	70.322	**0**	4.497	4.497	4.497

**Note.** The computation costs is the sum of computation time and communication time. Also, we implement $F_{\mathrm{eql}}$, $F_{\mathrm{abs}}$, and $F_{\mathrm{trn}}$ for SOCI^+^, SOCI, and POCF by using their own underlying protocols and the idea of TRUST.

Moreover, as shown in Table 2, TRUST demonstrates unique strengths in terms of communication costs across all protocols compared to existing solutions. This can be attributed to the fact that our experiments simulate real-world LAN communication, where frequent serialization and deserialization during data transfer between different processes introduce additional performance overhead. These factors increase communication latency, consume more bandwidth, and can cause network congestion, all of which negatively impact overall system throughput and the efficiency of homomorphic encrypted computations. Thanks to TRUST’s architecture, inter-process communication is avoided and only minimal context switching is required, eliminating such issues.

**Fig. 4:**
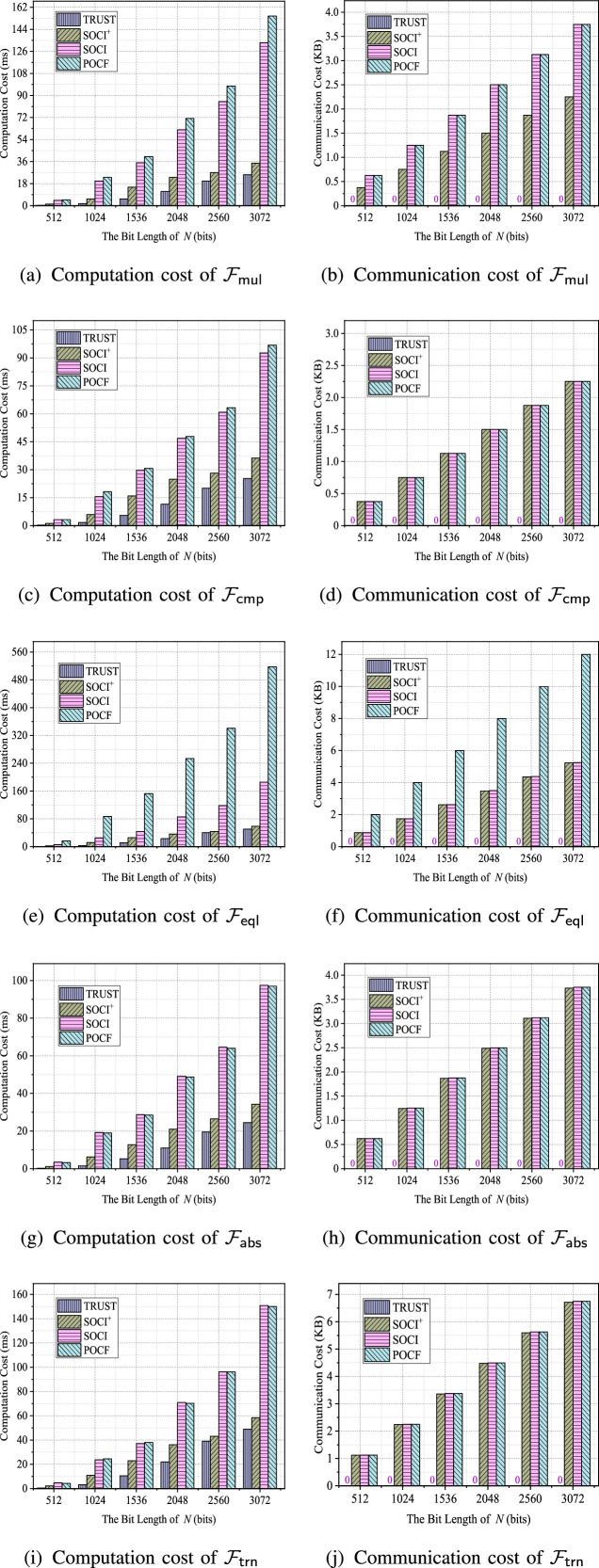
**Comparison of computation and communication costs for different schemes with a varying bit-length**N (ℓ=32,σ=128).

Fig. 4 presents a comprehensive analysis of the computation and communication costs of the proposed protocols across varying bit-lengths of N. TRUST exhibits significant advantages, particularly for the most widely used protocols such as $F_{\mathrm{mul}}$ and $F_{\mathrm{cmp}}$, both requiring only about 12 ms at |N|=2048. Furthermore, the communication costs across all protocols in TRUST remain constant at 0, regardless of the increase in N. Arguably, TRUST outperforms related solutions in terms of both computation and communication costs, suggesting that TRUST would offer significant advantages in high-performance computing and secure data processing applications.

### 8.4 SEAT Evaluations

We evaluate SEAT on a secure linear regression (SLR) training task. The scenario involves a data seller holding the dataset (X, $\vec{y}$), a data buyer providing the initial model parameters ($\vec{w}$, b, η), and a single TEE-equipped server acting as a data broker. The workflow proceeds as follows: 1) The seller and the buyer encrypt their respective inputs (i.e., ⟦X⟧, $⟦\vec{y}⟧$ and $⟦\vec{w}⟧$, ⟦b⟧, ⟦η⟧, respectively) before submitting the ciphertexts to the broker. 2) The broker performs SLR training using the encrypted data and returns the results ($⟦{\vec{w}}^{\prime }⟧$, $⟦b^{\prime }⟧$) upon completion of the training. 3) The buyer obtains the decrypted results ${\vec{w}}^{\prime },b^{\prime }$. We select SLR because its core operations, primarily multiplications over ciphertexts, are well-supported by the proposed TRUST. Furthermore, TRUST’s practical SOC framework and its support for other secure operations via threshold decryption and masking techniques provide a solid foundation for the practical implementation of SEAT.

To validate the effectiveness of the proposed SEAT, we compare it with SDTE [37] and Baseline. In particular, SDTE is configured to use an AES-256 symmetric encryption scheme to protect all data entering and exiting the enclave, consistent with its original specification. Baseline without any data protection serves as a performance reference. The experiments are conducted on two public datasets, i.e., Student Performance^4^ and Fish Market^5^, using three evaluation metrics MSE, $R^{2}$ and MAE.

**Fig. 5:**
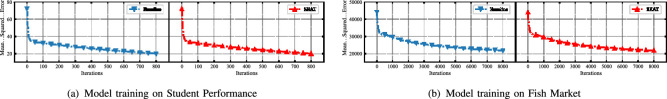
**Gradient descent performance of Baseline and SEAT across iterations**.

As shown in Table 3, SEAT achieves identical MSE, $R^{2}$ and MAE scores as both Baseline and SDTE at each training iteration, indicating that they result in the same final model. Furthermore, Fig. 5 intuitively shows that the convergence trajectory of SEAT perfectly matches that of the unprotected Baseline during SLR training. Based on these results, we argue that SEAT is correct, stable, and entirely feasible.

**Table 3:** **Cross-Dataset Feasibility Study and Performance Assessment of Baseline, SDTE, and SEAT**.

Dataset	Scale	Settings	Iterations	Baseline			SDTE [37]			SEAT		
				MSE	$R^{2}$	MAE	MSE	$R^{2}$	MAE	MSE	$R^{2}$	MAE
Student Performance	10000×6	learning rate (η): 0.0001	200	30.072	0.917	4.439	30.072	0.917	4.439	30.072	0.917	4.439
		bias (b): 0.0	400	26.167	0.928	4.135	26.167	0.928	4.135	26.167	0.928	4.135
		weights ($\vec{w}$): 5 dimensions, all 0	600	22.904	0.937	3.866	22.904	0.937	3.866	22.904	0.937	3.866
		batch size: 64	800	20.140	0.944	3.622	20.140	0.944	3.622	20.140	0.944	3.622
Fish Market	159×7	learning rate (η): 0.0001	2000	27063.899	0.739	136.322	27063.899	0.739	136.322	27063.899	0.739	136.322
		bias (b): 0.0	4000	24382.241	0.765	129.318	24382.241	0.765	129.318	24382.241	0.765	129.318
		weights ($\vec{w}$): 6 dimensions, all 0	6000	22908.562	0.779	124.621	22908.562	0.779	124.621	22908.562	0.779	124.621
		batch size: 16	8000	21927.701	0.788	121.025	21927.701	0.788	121.025	21927.701	0.788	121.025

**Remark.** The training and test sets each comprise half of their total sample count. $\text{MSE}=\frac{1}{n}\sum_{i=1}^{n}{\left(y_{i}-{\hat{y}}_{i}\right)}^{2}$, $R^{2}=1-\frac{\sum_{i=1}^{n}{\left(y_{i}-{\hat{y}}_{i}\right)}^{2}}{\sum_{i=1}^{n}{\left(y_{i}-\bar{y}\right)}^{2}}$,

$\text{MAE}=\frac{1}{n}\sum_{i=1}^{n}\left|y_{i}-{\hat{y}}_{i}\right|$ ($y_{i}$: True Value, ${\hat{y}}_{i}$: Predicted Value, $\bar{y}$: Mean of True Values).

## 9 Conclusion

In this work, we proposed TRUST, a toolkit for TEE-assisted secure outsourced computation over integers by seamlessly integrating a (2,2)-threshold Paillier cryptosystem and TEE. TRUST avoids any collusion attacks from a twin-server architecture of SOC. TRUST not only enriches computational operations, but also improves their performance. Also, we designed a secure data trading solution SEAT based on the proposed TRSUT and confirmed its feasibility. For future work, we will explore TRUST to more complex computation tasks, such as privacy-preserving machine learning inference.

## CRediT authorship contribution statement

**Bowen Zhao:** Conceptualization, Methodology, Writing – original draft, Writing – review & editing. **Jiuhui Li:** Software. **Cheng Qiao:** Project administration. **Jia-Nan Liu:** Formal analysis, Methodology. **Qingqi Pei:** Supervision. **Yulong Shen:** Funding acquisition, Investigation.

## Declaration of competing interest

The authors declare that they have no conflicts of interest in this work.

## Footnotes

1 TRUST: a toolkit for **T**EE-assisted secu**R**e o**U**t**S**ourced compu**T**ation over integers

2 SEAT: SEcure data trAding based on TRUST

3 This work has been open-sourced and can be accessed at: https://github.com/XidianSCgroup/TRUST

4 This dataset is available at: https://www.kaggle.com/datasets/nikhil7280/student-performance-multiple-linear-regression

5 This dataset is available at: https://www.kaggle.com/datasets/vipullrathod/fish-market
